# Accelerated Aging Effects Observed In Vitro after an Exposure to Gamma-Rays Delivered at Very Low and Continuous Dose-Rate Equivalent to 1–5 Weeks in International Space Station

**DOI:** 10.3390/cells13201703

**Published:** 2024-10-15

**Authors:** Juliette Restier-Verlet, Mélanie L. Ferlazzo, Adeline Granzotto, Joëlle Al-Choboq, Camélia Bellemou, Maxime Estavoyer, Florentin Lecomte, Michel Bourguignon, Laurent Pujo-Menjouet, Nicolas Foray

**Affiliations:** 1 INSERM U1296 Unit “Radiation: Defense, Health, Environment”, 28 Rue Laennec, 69008 Lyon, France; juliette.restier--verlet@inserm.fr (J.R.-V.); melanie.ferlazzo@gmail.com (M.L.F.); adeline.granzotto@inserm.fr (A.G.); joelle.al-choboq@inserm.fr (J.A.-C.); camelia.bellemou@hotmail.com (C.B.); michel.bourguignon@inserm.fr (M.B.); 2 Universite Claude Bernard Lyon 1, CNRS, Ecole Centrale de Lyon, INSA Lyon, Université Jean Monnet, ICJ UMR5208, Inria, 69622 Villeurbanne, France; maxime.estavoyer@inria.fr (M.E.); florentin.lecomte@etu.univ-lyon1.fr (F.L.); pujo@math.univ-lyon1.fr (L.P.-M.); 3 Département de Biophysique et Médecine Nucléaire, Université Paris Saclay, Versailles St. Quentin-en-Yvelines, 78035 Versailles, France

**Keywords:** space radiation, astronauts, risks, DNA double-strand breaks, accelerated aging, ATM protein

## Abstract

Radiation impacting astronauts in their spacecraft come from a “bath” of high-energy rays (0.1–0.5 mGy per mission day) that reaches deep tissues like the heart and bones and a “stochastic rain” of low-energy particles from the shielding and impacting surface tissues like skin and lenses. However, these two components cannot be reproduced on Earth together. The MarsSimulator facility (Toulouse University, France) emits, thanks to a bag containing thorium salts, a continuous exposure of 120 mSv/y, corresponding to that prevailing in the International Space Station (ISS). By using immunofluorescence, we assessed DNA double-strand breaks (DSB) induced by 1–5 weeks exposure in ISS of human tissues evoked above, identified at risk for space exploration. All the tissues tested elicited DSBs that accumulated proportionally to the dose at a tissue-dependent rate (about 40 DSB/Gy for skin, 3 times more for lens). For the lens, bones, and radiosensitive skin cells tested, perinuclear localization of phosphorylated forms of ataxia telangiectasia mutated protein (pATM) was observed during the 1st to 3rd week of exposure. Since pATM crowns were shown to reflect accelerated aging, these findings suggest that a low dose rate of 120 mSv/y may accelerate the senescence process of the tested tissues. A mathematical model of pATM crown formation and disappearance has been proposed. Further investigations are needed to document these results in order to better evaluate the risks related to space exploration.

## 1. Introduction

Exposure to ionizing radiation (IR) is one of the major hazards for astronauts [1,2,3,4]. However, in order to reliably quantify the space radiation-induced (RI) risks for astronauts, the sources of space radiation should be rigorously characterized and the tissues at risk well identified.

Our systematic analysis of the dosimetry data of all the Russian and American space civil missions, from Gagarin’s one in 1962 to the last Space Shuttle in 2011 [5], has revealed a constant doserate of about 0.28 ± 0.17 mSv per space mission day (i.e., 102 ± 62 mSv/y), in agreement with more recent literature and calculations [6,7]. Such a continuous “bath” of IR is not naturally reproducible at the surface of Earth. Indeed, its doserate is higher than the highest doserate received on average by inhabitants of the highest radiation background area (HBRA), Ramsar, in Iran, i.e., about 45 mSv/y [8,9,10,11]. This bath of high-energy rays was found to be essentially composed of photons [5,12]. The relative contribution of neutrons from 0.1 to 1000 MeV in this bath of IR represents a few percent of the contribution of photons [13]. Similarly, the contribution to the dose of heavy ions belonging to the galactic component radiation was found to be negligible as well [14,15]. It is noteworthy that the doserate of the “bath of radiation” is also of the same order as the radiation backgrounds assessed on the surface of the Moon and Mars (110–380 and 130–260 mSv/y, respectively), although the composition of radiation may be different [16,17,18,19,20]. In addition to the bath of IR, astronauts may be exposed to a “stochastic rain” of low-energy particles or ions resulting from the interactions between space radiation and shielding. Interestingly, such low-energy particles deliver all their energy in surface tissues like skin or lenses. By contrast, the bath of high-energy rays likely impacts deeper tissues like bones and the heart. Skin, lens, bones, and heart can be considered as the major human tissues at risk for space exploration [21,22].

Exposure to IR can have three major clinical consequences: RI toxicity (or radiosensitivity), RI cancer (radiosusceptibility), and RI accelerated aging (radiodegeneration) [23]. The occurrence of each of these consequences strongly depends on the IR dose, the irradiation scenario, and the tissue exposed. For example, RI toxicity requires IR doses higher than Gy and has never been described in the frame of space missions, even after solar flares [24]. Conversely, the occurrence of RI cancer or accelerated aging features requires sublethal doses that can theoretically be met in space missions. While no convincing cancer incidence has been described in the cohort of astronauts [25,26], 68% of astronauts show cataracts, a result of RI-accelerated aging of lens cells [27,28]. However, reliable and specific molecular biomarkers of aging remain to be determined to better quantify the accelerated aging risk. In 2023, in the frame of our experiments on Alzheimer’s disease (AD), we observed that the ataxia telangiectasia mutated protein (ATM) kinase protein, a DNA damage sensor, localizes around the nucleus of skin AD fibroblasts by forming a perinuclear crown of ATM [29]. We have shown that, in response to permanent stress, DNA dimers progressively monomerize, allowing the diffusion of monomers to the nucleus. Some overexpressed cytoplasmic proteins called X-proteins, specific to each tissue and individual, interact with ATM to form ATM-X-protein complexes. Thereafter, ATM monomers cumulate all around the nucleus and re-associate to form layers of ATM dimers [29]. In the case of AD fibroblasts, one of the X-proteins that participates in the formation of the perinuclear ATM crown was found to be apolipoprotein E (APOE). Interestingly, observations in cells from other aging syndromes and tissues have also revealed perinuclear ATM crowns and consolidated the specificity of such biomarkers for the detection of the accelerated aging process [30].

In this study, we aimed to investigate the molecular consequences of exposure of human tissues identified at risk for space exploration (skin, bones, lens, heart) to gamma-rays at a low and continuous dose rate equivalent to 1–5 week missions in the International Space Station (ISS). To this aim, we have accumulated radiobiological data by exposing cells at the radiation MarsSimulator facility (Toulouse University, France), which delivers gamma-rays at 0.33 +/− 0.17 mSv/day (120 mSv/year) compatible with the values evoked above [31].

## 2. Materials and Methods

### 2.1. Cell Lines

All experiments involved human untransformed cell lines cultured as monolayers in standard cell culture conditions reported elsewhere. The cellular characteristics of the cells used in this study are detailed in Table 1. Hs27, a radioresistant cell line originating from an apparently healthy individual, served as a control [32]. Heart myocytes, heart fibroblasts, and lens epithelial cell lines were purchased from ScienCell research laboratories (Carlsbad, CA, USA); the skin fibroblast GM03399 cell lines deriving from an *ATM^+/^^−^* mutated donor were purchased from Coriell Institute (Camden, NJ, USA), and all the other cell lines belong to the COPERNIC collection of the lab. The COPERNIC cell lines were obtained with all sampling protocols approved by the national ethical committee. The resulting cells were declared to the Ministry of Research under the numbers DC2008-585, DC2011-1437, and DC2021-3957. The radiobiological data of the COPERNIC collection are protected under reference IDDN.FR.001.510017.000.D.P.2014.000.10300 [32] (Table 1).

### 2.2. X-ray Irradiation at MarsSimulator Facility

Irradiations at the MarsSimulator facility were performed in cooperation with the Pr. Monique Courtade-Saïdi research team at the Cancer Research Center of Toulouse (CRCT, Toulouse, France). The continuous irradiation was provided by thorium nitrate salts (Th(NO_3_), 4×H_2_O), a crystalline salt. The natural thorium is almost 100% ^232^Th, so the features of thorium radioactivity emission are dominated by the decay characteristics of ^232^Th. At the radioactive equilibrium, ^232^Th emits alpha-rays whose energy ranges from 4 to 9 MeV, β^−^-rays whose Emax ranges from 600 keV to 2 MeV, and γ-rays whose energy ranges from 200 keV to 3 MeV [31]. The thorium nitrate has a half-life of 1.405 × 10^10^ years, which allows an invariable irradiation for the duration of the experiment. The thorium nitrate powder was contained in three layers of airtight plastic bags (33 × 36.5 cm with a total thickness of 0.4 to 1.2 cm) and enclosed in a protective cardboard envelope (32 × 44 cm), itself covered with an airtight plastic bag. This device, considered a sealed source of γ-rays, was placed in a cell incubator. All the other characteristics of the MarsSimulator facility have been described previously [31]. Cells were placed 14 cm above the irradiation for a duration of three weeks at a dose rate of about 13.7 μSv/h, which corresponds to about 120 mSv/y.

### 2.3. Immunofluorescence Assay and Micronuclei Assay

The immunofluorescence protocol and nuclear foci scoring were performed as described elsewhere [33]. The foci scoring procedure was applied in the frame of the Soleau envelope and patents (FR3017625 A1, FR3045071 A1, EP3108252 A1). More than 50 nuclei were analyzed per experiment and per post-irradiation time, with three independent replicates performed. Inter-reader foci scoring revealed no significant difference, whether performed manually or by computerized ImageJ v1.5 or Olympus foci scoring software (v2.0). During each immunofluorescence experiment, the 4′,6-diamidino-2-phenylindole (DAPI) counterstaining permits the identification of the nucleus and the quantification of micronuclei [34]. The micronuclei yield assessed may not be numerically equivalent to that obtained with the micronucleus assay involving cytochalasin B, but the protocol applied facilitates the analysis of the relationship between nuclear foci and micronuclei [35,36].

### 2.4. Statistical Analysis

Data were obtained from the indicated number of independent experiments and expressed as the mean ± standard error of the mean (SEM). Statistical analyses were performed using PRISM software version 9.5.1 (GraphPad Software, San Diego, CA, USA) or Kaleidagraph version 4.5.4 (Synergy Software, Reading, PA, USA).

## 3. Results

As detailed in Section 2, several untransformed human cell lines from different tissues identified at risk for space exploration (skin, lens, bones, heart) were exposed to ^232^Th salts embedded in plastic bags placed in a standard cell culture incubator (37 °C, 5% CO_2_, humid atmosphere) for 1 to 5 weeks, corresponding to 2.3 to 11.53 mSv. The yields of micronuclei and DSB assessed by γH2AX or by pATM immunofluorescence were used as molecular endpoints [33].

### 3.1. Micronuclei

A micronucleus results from a DNA fragment after a chromosome break that remains unrepaired after mitosis [23]. The number of micronuclei reflects genomic instability and has been associated with an abnormal response to stress [37]. With regard to the spontaneous micronuclei, the yield of micronuclei assessed in the radioresistant Hs27 cells was found to be similar to that assessed in unirradiated cells referenced in the lab (0.7 ± 0.3 micronuclei per 100 cells), a numerical value consistent with our historical data (Table 2) [37]. With regard to the radiation-induced micronuclei, the yield of micronuclei did not vary significantly with the duration of exposure (*p* > 0.6), which is consistent with the range of doses delivered [32] (Table 2). In all other cell lines tested, the yield of spontaneous micronuclei was higher than controls (*p* < 0.05), and the yields of micronuclei did not vary significantly with the duration of exposure (*p* > 0.6), suggesting that the application of a low and continuous dose rate of 120 mSv/year does not significantly increase genomic instability, in terms of micronuclei formation in a dose-dependent manner, whatever the tissue tested (Table 2).

### 3.2. DSB with γH2AX Immunofluorescence

**Figure 1:**
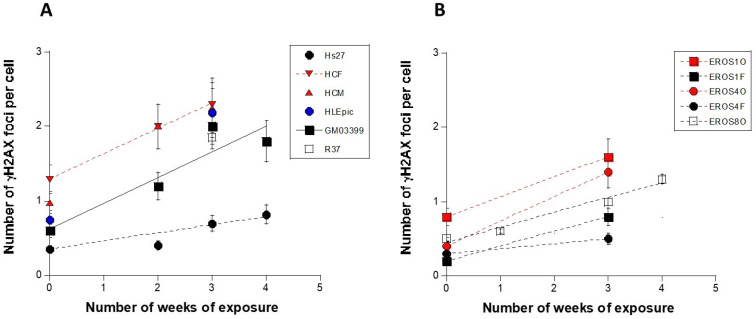
Number of γH2AX foci per cell was assessed by immunofluorescence in the indicated cell lines and at the number of weeks of exposure at 120 mSv/y. The panel (**A**) shows the data obtained from non-bone cells while the panel (**B**) shows the data obtained from bone cells only. Each plot corresponds to the mean of three replicated ± SEM. Each week represents an exposure to 2.3 mSv.

In response to IR, the ATM kinase is activated by monomerization; the monomers diffuse to the nucleus and phosphorylate the X variant of the H2A histone (γH2AX) at the DSB sites; such a step is accompanied by the formation of immunofluorescence γH2AX foci. Although ATM is not the only kinase that phosphorylates H2AX, ATM is required for the formation of early γH2AX foci [38,39]. The number of γH2AX foci per cell observed in the radioresistant control cell line before irradiation (0.34 ± 0.3 γH2AX foci per cell) was in agreement with the literature and our historical data [23,32]. The number of γH2AX foci increases moderately with the duration of the exposure (Figure 1; Table 3). Such an increase was consistent with a DSB induction rate of 37 ± 4 DSB per Gy per cell documented in our collection of radioresistant fibroblasts [32]. The slopes of the γH2AX foci as a function of dose were found to be 1.1 to 3 times higher than that obtained with radioresistant controls, suggesting the production of additional DSB as far as dose increases and an impairment of the DSB repair by comparison to the control data (Figure 1; Table 3).

If one considers the tissue dependence of the number of the γH2AX foci per cell as a function of dose, it is interesting to note that the bone cells showed 1.6 times more DSB than controls. This ratio reached 2 for the radiosensitive skin fibroblast cell lines (from ATM^+/−^ and NF1^+/−^ patients) and the heart myocytes. For the heart fibroblasts and the lens cells, the ratio reached 2.6 and 3, respectively. Lastly, the EROS skin lines showed different radiosensitivity, but the osteoblasts remained systematically more radiosensitive than their fibroblast counterparts (*p* < 0.5) (Table 3 and Table 4).

### 3.3. Observations with pATM Immunofluorescence

In a previous study, we demonstrated that the number of γH2AX foci is systematically twice as high as that of pATM foci [35]. Here, the number of pATM foci was assessed in all the situations described above. However, the number of γH2AX foci was so low that the number of pATM foci per cell was not measurable in nearly all the scenarios.

**Figure 2:**
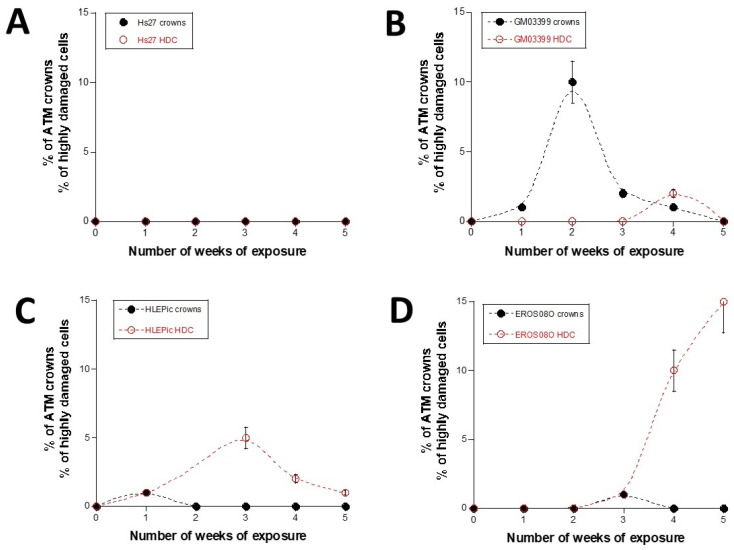
Percentage of ATM crowns and of highly damaged cells (HDC) observed in the indicated cell lines at each week of exposure to 120 mSv/y ((**A**): radioresistant skin fibroblasts; (**B**): radiosensitive skin fibroblasts; (**C**): lens cells; (**D**): bone cells). Each plot corresponds to the mean of three replicated ± SEM. Each week represents an exposure to 2.3 mSv.

**Figure 3:**
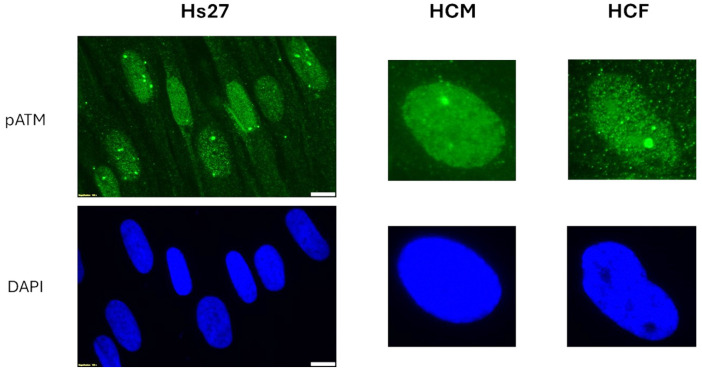
Representative immunofluorescence images of pATM foci (green) and DAPI-counterstained ones (blue) were observed in the indicated cells during the first week of exposure to 120 mSv/y. Each week represents an exposure to 2.3 mSv. The white bar represents 10 µm.

**Figure 4:**
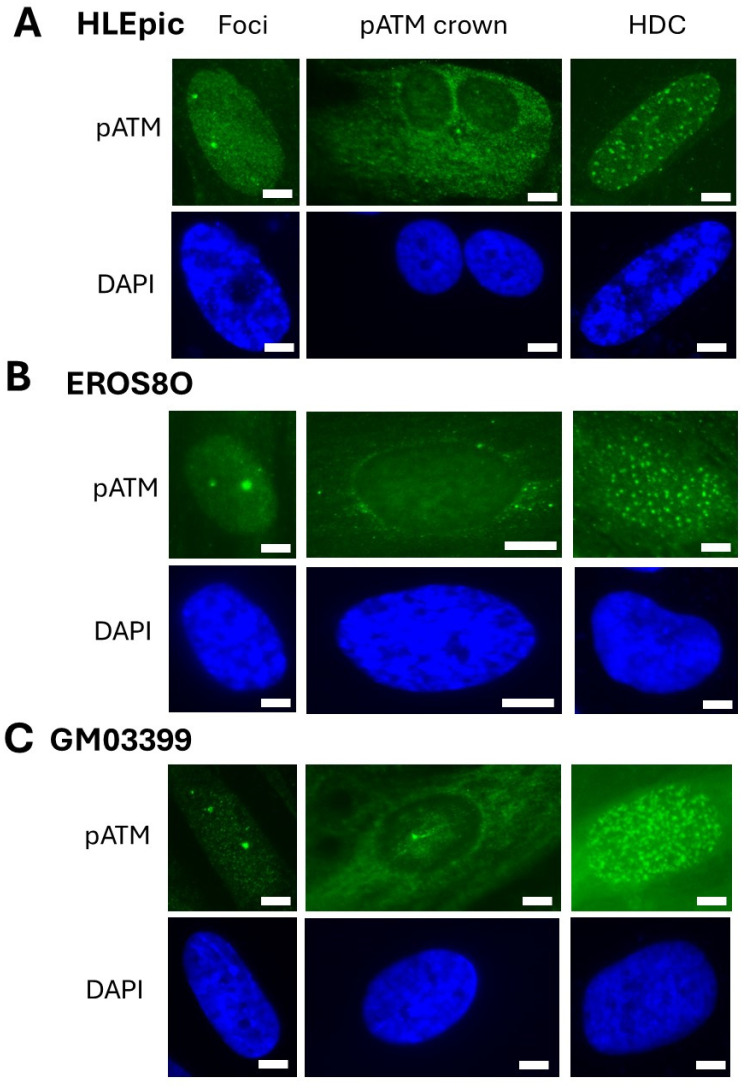
Representative immunofluorescence images of pATM foci and pATM crowns (green) were observed in the indicated cells at the 1st week (HLEpic and GM03399) or the 3rd (EROS08O) week of exposure to 120 mSv/y and of HDC at the (**A**) 1st (HLEpic), (**B**) 3rd (EROS08O), and (**C**) 4th week (GM033999). Each image has been counterstained with DAPI (blue). The white bar represents 5 µm.

Interestingly, for the lens, bone cells, and cells from the radiosensitive skin, we observed a perinuclear localization of the pATM in some cells (Figure 2, Figure 3 and Figure 4). In 2023, we pointed out an accelerated aging-specific marker based on the ATM proteins: in response to low and continuous stress, the ATM monomers that diffuse to the nucleus meet, before the nuclear membrane, some overexpressed perinuclear proteins (called X-proteins because they can be specific to the tissue, the individual, and/or the stress) [40,41]. In most aging syndromes, the X-proteins associated have been found to be abnormally overexpressed in the immediate periphery of the nucleus [29,30]. Consequently, the ATM monomers attached to the X-proteins will aggregate around the nucleus and form a perinuclear pATM crown, blocking the entry of DNA damage signaling and repair proteins in the nucleus [29]. During this process, DNA strand breaks accumulate in the nucleus and increase and accelerate the senescence process [42,43]. Such observation has also been conducted in lens cells undergoing cataracts [30]. Here, in our conditions of irradiation, pATM crowns were observed in lens and radiosensitive skin at the 1st week (i.e., 2.3 mSv) and in bone cells at the 3rd week of exposure at 120 mSv/y (i.e., 6.9 mSv).

Interestingly, after the week of their appearance, the pATM crowns disappeared, and some cells with larger nuclei containing pATM or γH2AX foci were observed. The potential link between pATM crowns and such highly damaged cells (HDC) will be discussed in the Section 4 (Table 4, Figure 2, Figure 3 and Figure 4).

## 4. Discussion

### 4.1. From the Tissues Identified at Risk for Space Exploration to the Number of DSB

Thanks to access to the MarSimulator facility, we were able to expose four major types of tissues identified as being at risk for space exploration (skin, lens, bones, and heart) to IR. By excluding the tissue reactions of toxicities linked to exposure to doses higher than 2 Gy (never reached in space), the consequences of sublethal exposure to radiation in space may be RI cancer or else RI accelerated aging [44,45].

Since low-energy particles target surface tissues, RI skin and eye tumors (i.e., melanoma and retinoblastoma) may be evoked [25,45,46]. Interestingly, an increase in mortality from skin melanoma has been reported among astronauts [47]. However, this increase was hypothesized to be consistent with the exposure to UV while astronauts are not subjected to higher exposure to UV in their spacecraft: such cancer origin may be, therefore, more dependent on lifestyle rather than occupational conditions, inasmuch as the time spent in a space mission is negligible in comparison with that spent on Earth, exposed to natural UV [48,49]. Regarding eye melanoma, to our knowledge, neither choroid melanoma nor retinoblastoma has been reported in astronauts. Regarding RI cancers potentially caused by high-energy photons, leukemia is the most probable RI cancer [50]. However, again, such a statement originated from Hiroshima bomb survivors’ data that concerned flash irradiation rather than continuous and low-dose-rated exposure [51]. No RI leukemia has been reported yet in astronauts; the risk of RI leukemia remains theoretical in space mission conditions but may be evoked after missions longer than 1 year. This short review reveals that, whether they affect deep or surface tissues, the occurrence of RI cancers is possible but is conditioned to the long duration of space missions [25,52].

Radiation-induced degeneration has been defined as the proneness to RI-accelerated aging of specific tissues, generally attributable to the tolerance of some unrepaired DNA breaks [23]. The most documented radiodegeneration reactions are RI cataracts. To date, they appear as the most probable consequence of exposure to space radiation [53]. Forty-eight cases of severe lens opacification (16.2%) were observed among the 295 NASA astronauts who participated in the LSAH (Longitudinal Study of Astronaut Health) study, but 86% of the astronauts who stayed in space suffered from a pathology of the eye [27,54,55,56]. Throughout history, the risk of RI cataracts has been underestimated. Indeed, the annual occupational dose limits to the eye have decreased regularly all along the international radioprotection recommendations: from 300 mSv in 1977 to 150 mSv in 1984, and, to date, 20 mSv per year averaged over five years (100 mSv for 5 years, with no single year exceeding 50 mSv) [57]. The fact that the last recommended threshold dose values (20 mSv per year) are much lower than the average doserate for space missions (120–146 mSv per year) is, therefore, consistent with a significant occurrence of RI cataracts reported in astronauts. In addition to the RI cataracts, some astronauts of the Skylab, Soyuz, Apollo, and ISS missions were also shown to suffer from spontaneous light flashes (LF) visually perceived. The RI nature of such events has been evoked, and their occurrence may be compatible with the impact of the low-energy particles on the eye. However, further investigations are needed to establish the causal links between the dose, the radiation type, and the LF occurrence [58,59,60,61,62]. Lastly, while skin represents the essentials of the surface of the human body, there is no evidence of any RI-accelerated aging in astronauts’ skin. However, even if the lens represents a very small but unprotected target surface, it must be stressed that skin is essentially covered by astronauts’ worksuits. However, worksuits do not protect significantly from the predominant bath of high-energy photons but may limit the effect of low-energy particles. Further investigations are therefore needed to reply to the questions raised by the occurrence of RI cataracts and the absence of obvious agingeffects on the skin.

In addition to cataracts and eye pathologies, loss of bone mass, currently described after space missions [63,64,65,66], may be another consequence of RI-accelerated aging. While the loss of bone mass has long been attributed to microgravity [65], some emerging data suggest that radiation may also affect bones [64]. Interestingly, the problem of bones gathers two radiobiological effects evoked above (1) in certain conditions (radiation type, dose, tissue type, number of cells irradiated); irradiated cells can emit Ca^2+^ ions flux in the extracellular medium, generally in proportion to the dose and of their content in calcium. Such radiation-induced Ca^2+^ release can reach the unirradiated cells in the close vicinity of the irradiated ones and produce an excess of genotoxic stress. Such a phenomenon is called the radiation-induced bystander effect [67]. Since bones are rich in calcium, they may be characterized by an enhanced Ca^2+^-dependent bystander effect. (2) Bone cells appear to be more radiosensitive than skin cells [68].

Lastly, the cardiovascular system may belong to the group of tissues at risk for space exploration. This hypothesis is notably motivated by the observation that irradiation may increase the risk of heart attack. However, similarly to the eye, such observations originate from the follow-up of breast cancer women treated by radiotherapy, i.e., from high and fractionated doses (more than 50% of patients elicit a significant risk of heart attack for 10 years post-radiotherapy against breast tumor [69,70,71]). However, there are a few data about cardiovascular system aging after continuous and low-dose-rated irradiation. Furthermore, there is still no evidence of any cardiovascular disease in astronauts that would be caused by exposure to space radiation [72,73].

Altogether, this short review of the state-of-the-art suggests that space radiation may cause accelerating aging to the ocular, bone, and maybe cardiovascular systems, but the threshold dose above which the risk may be significant needs to be determined. The question of radiodegeneration has been recently raised by a unique space experiment with two twins [74,75,76,77,78]. During a one-year ISS mission, one male twin was on board while his monozygotic twin served as a genetically matched ground control. Telomeres were found longer in the twin who was on board during spaceflight and shortened rapidly upon return to Earth. An increase in the number of chromosomal inversions was also found after spaceflight [77].

Anti-*γH2AX* immunofluorescence reveals DSB managed by the non-homologous end-joining (NHEJ) pathway, the most predominant DSB repair pathway in humans. At a very low doserate, like 120 mSv/y, the incidence of about 40 DSB/Gy for radioresistant skin cell lines was respected, suggesting that the DSB repair rate may not be dependent on the dose rate. Conversely, for more radiosensitive cells, impairments of DSB recognition and/or DSB repair increase the slope of the curves describing the number of DSB induced as a function of exposure time. The largest ratio reached with radioresistant cells was obtained with the lens. Interestingly, there is no direct link between the slope of the curve of DSB induction and the occurrence or intensity of pATM crowns and HDC. This finding suggests that the rate of DSB per unit of time is not necessarily the only factor to explain the formation of ATM crowns and HDC and, therefore, the accelerated aging features observed in these cell lines.

### 4.2. From the ATM Crowns to the HDC Cells

**Figure 5:**
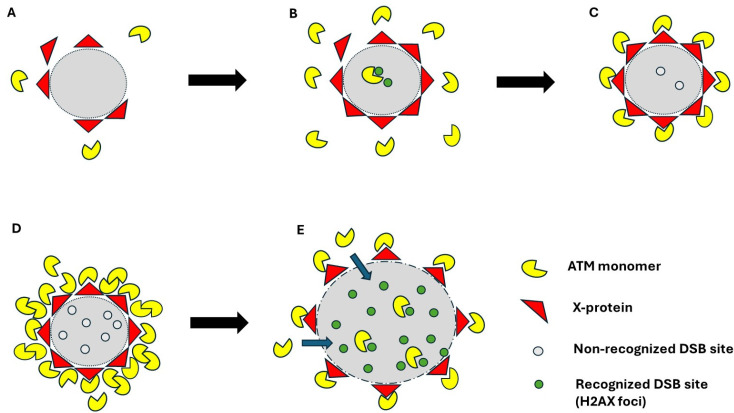
Hypothetic model of formation of pATM crowns and HDC. When permanent stress is applied to cells showing a perinuclear overexpression of an X-protein (**A**), progressively, the ATM monomers bind to X-proteins. At this step, since ATM monomers can enter the nucleus, DSB recognition and repair are still possible (**B**). When the first layer of the pATM crown is complete, DSB recognition and repair are impossible (**C**). Since ATM monomers cumulate around the nucleus, such a high concentration of ATM monomers leads to the re-dimerization of ATM (**D**). The DNA strand breaks cumulate in the nucleus, and the chromatin decondenses and enters some ATM monomers that recognize the numerous DSB by forming γH2AX and ATM foci: the cell becomes HDC (**E**).

In the frame of the RIANS model, in response to sublethal and continuous stress, the ATM monomers diffuse to the nucleus and participate in the DSB recognition and repair. However, if X-proteins are overexpressed in cytoplasm, the flux of ATM monomers is reduced. As already reported [29], if the overexpressed X-proteins are perinuclear, some pATM crowns are progressively formed. We have shown that the number of cells showing pATM crowns obeys a sigmoidal function of time or cell passages in culture [29]. Indeed, as ATM dimer layers accumulate around the nucleus, any entry of ATM monomers into the nucleus becomes impossible: there are more and more DNA breaks in the nucleus, but less and less DNA damage is recognized. The literature reveals that, from a certain level of cumulative DNA breaks, the chromatin can be decondensed, and the nucleus size increases [79,80]. Interestingly, the size of the HDC cell nuclei observed after the pATM crown occurrence can be three times larger than that of cells showing ATM crowns. At this step, we can hypothesize that the sudden increase in the nucleus size of cells showing pATM crown would either disrupt the cohesive scaffold of the crowns or increase the nuclear pore size and permit some ATM monomers to enter the nucleus to reveal γH2AX and pATM foci reflecting the numerous DNA strand break sites. At the same time, some acidic release from the nucleus to the cytoplasm may induce the dissociation of ATM dimers, leading to the disappearance of the pATM crowns. Lastly, the DAPI staining clearly shows that HDC cells are not in G2/M but in G0/G1 cells (Figure 4 and Figure 5). In these conditions, bone or lens cells would be the most radiosensitive cell type among those tested here, showing an accelerated aging effect leading to bone loss or cataracts, currently observed even after the shortest space missions. Our observations would, therefore, be in agreement with clinical data [27,28,66,81]. Obviously, further investigations are needed to better document such an effect.

**Figure 6:**
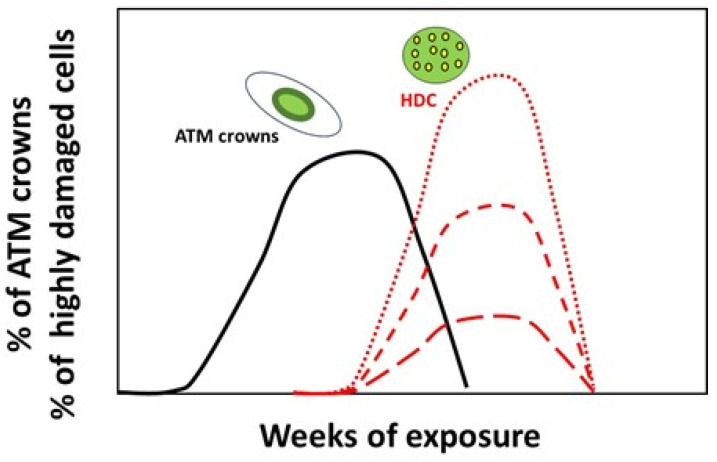
Theoretical illustration of the ATM crowns and HDC signals as a function of time. The occurrence of ATM crowns (in black) systematically precedes the formation of HDC (in red). However, the measurement may reveal much more or much less HDC than ATM crowns (the dotted or dashed lines reflect different response in the formation of HDC.

Interestingly, when the data shown in Figure 2 are analyzed, two major features emerge:

- The occurrence of ATM crowns always precedes the occurrence of HDC.
- The level of HDC observed may be either lower (like with the radiosensitive skin cells) or much higher than the “ATM crown signal” (like with the lens and bone cells) (Figure 2 and Figure 6).

If we expect that the fate of each ATM crown cell is to become one HDC, the two “ATM crowns” and “HDC” signals should be delayed in time but quantitatively equal. Maybe the lifetimes of ATM crowns and even of HDC are so short that a complete assessment of these two events is not possible. Hence, all the ATM crowns and HDC may not be instantaneously visible and quantifiable. Further investigations are therefore needed to better understand the link between ATM crowns and HDC occurrences and their clinical impact.

### 4.3. Mathematical Model

**Figure 7:**
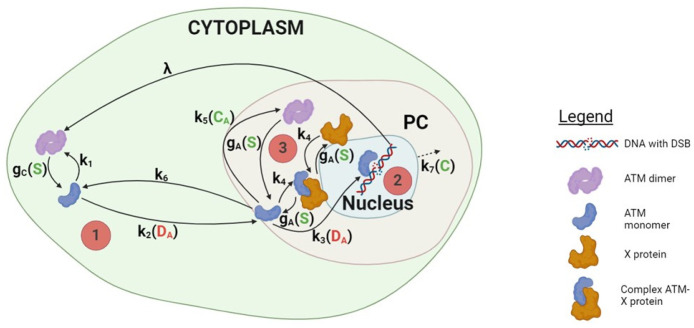
Schematic representation of the protein interaction described by our mathematical model. In the cytoplasm (light green background), ATM dimers are monomerized by oxidative stress, and ATM monomers migrate near the nucleus (step 1 in the figure). ATM monomers may eventually cross the nucleus membrane and reach the nucleus (light blue background) to detect DSB and trigger their repair process (step 2). On their way to the nucleus, cytoplasmic ATM monomers may form ATM-X protein complexes as well as ATM dimers around the nucleus, leading to the formation of a perinuclear ATM crown (light red background) (step 3). When the perinuclear ATM crown is formed and thick enough, cytoplasmic ATM monomers cannot reach the nucleus anymore.

As a final step, we adapted a previously introduced compartment model from the article [82] to describe the multiprotein complex interactions and mechanisms involved in the formation of the perinuclear ATM crown, its resorption due to the oxidative stress, and the nucleus growth (a consequence of the accumulation of DNA strand breaks). This model consists of seven compartments: $D_{C}$, $M_{C}$, $M_{A}$, A , $C_{A}$, $D_{A}$, and $M_{N}\;$ (Figure 7). The variable $D_{C}$ represents the concentration of ATM dimers in the cytoplasm while $M_{C}$ represents the concentration of ATM monomers in the cytoplasm. The variable $M_{A}$ refers to ATM monomers in the nucleus vicinity, A stands for the X-protein, and $C_{A}$ represents ATM-X-protein complexes (forming the inner layer of the perinuclear ATM crown (PC)). The variable $D_{A}$ corresponds to the perinuclear ATM dimers, forming the outer layer of the PC. The variable $M_{N}$ represents ATM monomers inside the nucleus in charge of the DSB repair process. In addition to these compartments, we have incorporated the nucleus radius (R) variation (related indirectly to the changes in the nucleus volume due to the accumulation of DNA strand breaks) as well as the cytoplasmic stress (S) induced by this nucleus volume change. A schematic illustration of the multiprotein complexes interactions in the cell is given in Figure 7. 

The relationships shown in Figure 7 can be translated into the following mathematical system of non-linear differential equations (where the derivative term corresponds to the time-variation (for clarity, we wrote $D_{c}^{\prime }$ for $dD_{c}\left(t\right)/dt$, and so on for the other variables):

$$\left\{\begin{array}{c} \begin{array}{c} D_{C}\prime =\lambda -d_{0}D_{C}+0.5k_{1}M_{C}^{2}-g_{C}D_{C},\\ M_{C}\prime =-k_{1}M_{C}^{2}-k_{2}\left(D_{A}\right)M_{C}+2g_{C}D_{C},\\ M_{A}^{\prime }=k_{2}\left(D_{A}\right)M_{C}-k_{3}\left(D_{A}\right)M_{A}-k_{4}A\cdot M_{A}-k_{5}\left(C_{A}\right)M_{A}^{2}+2g_{A}\left(R,S\right)D_{A}+g_{A}\left(R,S\right)C_{A}, \end{array}\\ M_{N}\prime =k_{3}\left(D_{A}\right)M_{A}-d_{1}M_{N},\\ \begin{array}{c} A^{\prime }=-k_{4}A\cdot M_{A}+g_{A}\left(R,S\right)C_{A},\\ \begin{array}{c} C_{A}^{\prime }=k_{4}A\cdot M_{A}-g_{A}\left(R,S\right)C_{A},\\ \begin{array}{c} D_{A}^{\prime }=0.5k_{5}\left(C_{A}\right)M_{A}^{2}-g_{A}\left(R,S\right)D_{A},\\ R^{\prime }=r_{1}k_{7}\left(C\right)1_{C>K},\\ S^{\prime }=s_{1}k_{7}\left(C\right)1_{C>K}. \end{array} \end{array} \end{array} \end{array}\right.$$

in which $1_{C>K}$ indicates that the variations in R and S only begin once the threshold K is surpassed. We hypothesized that the perinuclear pATM crown should reach a sufficient thickness before the nucleus begins to expand. Note that a detailed explanation of each equation is given in the Supplementary Materials (see Supplementary Data).

**Figure 8:**
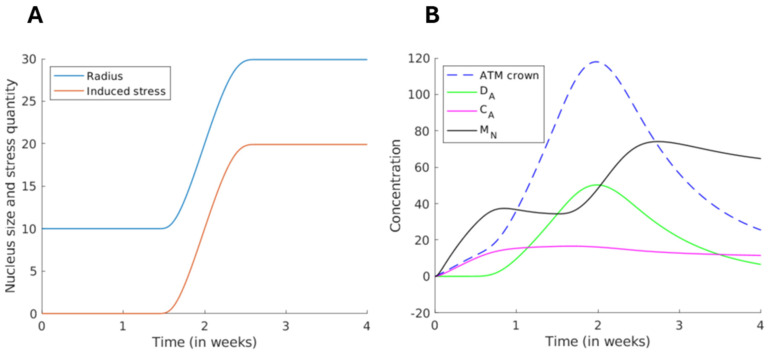
Numerical simulation representing the qualitative behavior of the different protein concentrations as a function of time. (**A**) We observed that as the perinuclear ATM crown (due to forced ATM concentration, in dashed blue curve) reaches a certain threshold (two weeks here), the radius of the nucleus (in blue curve) increases up to 3-folds its initial size, leading to the release of the induced stress (in orange curve). (**B**) These events lead to a monomerization of the ATM dimers (ATM dimers and ATM-X-protein–ATM complexes) in the crown (green and magenta curves). These events lead to the destruction of the pATM crown. In addition, the newly formed cytoplasmic ATM monomers may cross the nucleus and recognize the DSB (black curve).

The qualitative behavior of each protein involved in this model is shown in Figure 8. Until a critical threshold time is reached (here, the second week), the formation of the perinuclear ATM crown is observed. This step is explained by the increase in complexes $C_{A}$ and dimers $D_{A}$ formation. When the perinuclear ATM crown is completely formed, ATM monomers cannot reach the nucleus and cross its membrane. With continuous stress, DNA strandbreaks accumulate, which triggers the chromatin decondensation and causes an increase in the nucleus radius (up to 3 times larger than its original size). If the nucleus is too large, the nuclear membrane may facilitate the exchanges between the intranuclear and extranuclear liquids. Then, the protein trafficking in the nuclear membrane may be impaired, and ATM monomers can penetrate inside the nucleus: $M_{N}$ increases. Our calculations were stopped after four weeks. More quantitative data and further exposure times are needed to better detect the beginning of the HDC process.

## 5. Conclusions

By applying the continuous low-dose-rated exposure of IR to human tissues identified at risk for space exploration to mimic the irradiation reigning in the ISS, we observed the first molecular and cellular signs of RI senescence in bone, lens, and radiosensitive skin cells since the first weeks of the mission. Such molecular and cellular findings are in agreement with the clinical observations performed at the end of the most current mission (degenerative bone and cataracts). Such findings may be explained by the RIANS model, a mechanistic model based on the nucleoshuttling of the ATM protein, a very well-documented sensor of genotoxic stress. We have developed a mathematical model of the formation of pATM crowns, a specific marker of accelerated aging. Further investigations are needed to better document these steps and to develop specific countermeasures.

## Figures and Tables

**Table 1:** Major features of the cell lines used in this study.

Cell Line	Tissue Origin	Provider
Hs27	Radioresistant foreskin fibroblast	COPERNIC
GM03399	ATM+/− skin fibroblast	Coriell Institute
R37	NF1+/− skin fibroblast	COPERNIC
HCF	Heart fibroblast	ScienCell
HCM	Heart myocytes	ScienCell
HLEpiC	Lens epithelial cells	ScienCell
EROS01O	Osteoblast	COPERNIC
EROS01F	Skin fibroblast	COPERNIC
EROS04O	Osteoblast	COPERNIC
EROS04F	Skin fibroblast	COPERNIC
EROS08O	Osteoblast	COPERNIC

**Table 2:** Number of micronuclei per 100 cells.

Cell Line	Control	Week 1 (2.3 mSv)	Week 2 (4.6 mSv)	Week3 (6.9 mSv)	Week 4 (11.5 mSv)
Hs27	0.7 ± 0.3	1.9 ± 0.5	2.0 ± 0.5	2.1 ± 0.8	2.2 ± 0.5
GM03399	1.6 ± 0.5	3.0 ± 1.5	1.9 ± 1.5	2.0 ± 0.5	4.1 ± 2.0
HCF	1.4 ± 0.6	3.2 ± 0.5	4.2 ± 1.5	nd	4.1 ± 2.0
HCM	1.3 ± 0.5	2.0 ± 0.4	3.1 ± 1.5	2.4 ± 1.2	3.2 ± 1.5
EROS08O	1.2 ± 0.6	2.6 ± 0.5	3.0 ± 1.5	3.1 ± 1.5	2.0 ± 0.5
HLEpiC	2.0 ± 0.5	1.6 ± 0.8	2.8 ± 1.5	3.3 ± 1.2	2.5 ± 0.5

**Table 3:** Fitting data of the number of γH2AX foci as a function of dose.

Cell Line	Tissue Origin	Data Fit	R Coefficient
Hs27	Radioresistant foreskin fibroblast	y = 0.29 + 0.122x	R = 0.925
GM03399	ATM^+/−^ skin fibroblast	y = 0.62 + 0.342x	R = 0.925
R37	NF1^+/−^ skin fibroblast	y = 0.60 + 0.416x	R = 0.99
HCF	Heart fibroblast	y = 1.01 + 0.435x	R = 0.98
HCM	Heart myocytes	y = 1.29 + 0.339x	R = 0.99
HLEpiC	Lens epithelial cells	y = 0.75 + 0.476x	R = 0.99
EROS01O	Osteoblast patient EROS01	y = 0.8 + 0.266x	R = 0.99
EROS01F	Skin fibroblast patient EROS01	y = 0.2 + 0.20x	R = 0.99
EROS04O	Osteoblast patient EROS04	y = 0.4 + 0.33x	R = 0.99
EROS04F	Skin fibroblast patient EROS04	y = 0.3 + 0.16x	R = 0.99
EROS08O	Osteoblast patient EROS08	y = 0.45 +0.20x	R = 0.98

**Table 4:** Summary of the ATM features in the tissues tested.

Human Tissues	Average DSB/Gy	Ratio with Radioresistant Skin	Maximal % of ATM Crowns	First Crowns Observed at	Maximal % of HDC	First HDC Observed at
Radioresistant skin	0.16 ± 0.04	1	0	0	0	0
Bones	0.26 ± 0.07	1.6	1	3rd week (6.9 mSv)	15	3rd week (6.9 mSv)
Radiosensitive skin	0.37 ± 0.04	2	10	1st week (2.3 mSv)	2	4th week (9.2 mSv)
Heart myocyte	0.34 ± 0.07	2	0	0	0	0
Heart fibroblasts	0.43 ± 0.07	2.6	0	0	0	0
Lens	0.47± 0.07	3	1	1st week (2.3 mSv)	5	1st week (2.3 mSv)

## Data Availability

All the data can be provided at a reasonable request.

## Supplementary Materials

The following supporting information can be downloaded at: https://www.mdpi.com/article/10.3390/cells13201703/s1: Mathematical model; Table S1: Numerical values for all the parameters of the model.

## References

[1] Cucinotta F.A., Kim M.-H.Y., Ren L. (2005). Managing Luna and Mars Mission Radiation Risks. Part I; Cancer Risks, Uncertainties and Shielding Effectiveness.

[2] Durante M., Cucinotta F.A. (2008). Heavy ion carcinogenesis and human space exploration. Nat. Rev. Cancer.

[3] Durante M. (2014). New challenges in high-energy particle radiobiology. Br. J. Radiol..

[4] Cucinotta F.A., Schimmerling W., Wilson J.W., Peterson L.E., Badhmar G.D., Saganti P.B., Dicello J.F. (2011). Space Radiation Cancer Risk Projections for Exploration Missions: Uncertainty Reduction and Mitigation.

[5] Maalouf M., Durante M., Foray N. (2011). Biological effects of space radiation on human cells: History, advances and outcomes. J. Radiat. Res..

[6] Smith M.B., Khulapko S., Andrews H.R., Arkhangelsky V., Ing H., Koslowksy M.R., Lewis B.J., Machrafi R., Nikolaev I., Shurshakov V. (2016). Bubble-detector measurements of neutron radiation in the international space station: ISS-34 to ISS-37. Radiat. Prot. Dosim..

[7] Reitz G., Beaujean R., Benton E., Burmeister S., Dachev T., Deme S., Luszik-Bhadra M., Olko P. (2005). Space radiation measurements on-board ISS—The DOSMAP experiment. Radiat. Prot. Dosim..

[8] Mohammadi S., Taghavi-Dehaghani M., Gharaati M.R., Masoomi R., Ghiassi-Nejad M. (2006). Adaptive response of blood lymphocytes of inhabitants residing in high background radiation areas of ramsar- micronuclei, apoptosis and comet assays. J. Radiat. Res..

[9] Talebian H., Monfared A.S., Niaki H.A., Fattahi S., Bakhtiari E., Changizi V. (2020). Investigating the expression level of NF-KB and HIF1A genes among the inhabitants of two different background radiation areas in Ramsar, Iran. J. Environ. Radioact..

[10] Sohrabi M. Recent radiological studies of high level natural radiation areas of Ramsar. Proceedings of the International Conference on High Levels of Natural Radiation (ICHLNR).

[11] Bavarnegin E., Fathabadi N., Vahabi Moghaddam M., Vasheghani Farahani M., Moradi M., Babakhni A. (2013). Radon exhalation rate and natural radionuclide content in building materials of high background areas of Ramsar, Iran. J. Environ. Radioact..

[12] Restier-Verlet J., El-Nachef L., Ferlazzo M.L., Al-Choboq J., Granzotto A., Bouchet A., Foray N. (2021). Radiation on Earth or in Space: What Does It Change?. Int. J. Mol. Sci..

[13] Kohler J., Ehresmann B., Zeitlin C., Wimmer-Schweingruber R.F., Hassler D.M., Reitz G., Brinza D.E., Appel J., Bottcher S., Bohm E. (2015). Measurements of the neutron spectrum in transit to Mars on the Mars Science Laboratory. Life Sci. Space Res. Amst..

[14] Benton E.R., Benton E.V. (2001). Space radiation dosimetry in low-Earth orbit and beyond. Nucl. Instrum. Methods Phys. Res. Sect. B Beam Interact. Mater. At..

[15] Cucinotta F.A., Kim M.H., Willingham V., George K.A. (2008). Physical and biological organ dosimetry analysis for international space station astronauts. Radiat. Res..

[16] Straume T., Blattnig S., Zeitlin C., Levine J.S., Schild R.E. (2010). Radiation Hazards and the Colonization of Mars. The Human Mission to Mars: Colonizing the Red Planet.

[17] Reitz G., Berger T., Mattiae D. (2012). Radiation exposure to the Moon environment. Planet. Space Sci..

[18] Zeitlin C., Hassler D.M., Cucinotta F.A., Ehresmann B., Wimmer-Schweingruber R.F., Brinza D.E., Kang S., Weigle G., Bottcher S., Bohm E. (2013). Measurements of energetic particle radiation in transit to Mars on the Mars Science Laboratory. Science.

[19] Abbasi S., Mortazavi S.A.R., Mortazavi S.M.J. (2019). Martian Residents: Mass Media and Ramsar High Background Radiation Areas. J. Biomed. Phys. Eng..

[20] Zhang S., Wimmer-Schweingruber R.F., Yu J., Wang C., Fu Q., Zou Y., Sun Y., Wang C., Hou D., Bottcher S.I. (2020). First measurements of the radiation dose on the lunar surface. Sci. Adv..

[21] Hellweg C.E., Baumstark-Khan C. (2007). Getting ready for the manned mission to Mars: The astronauts’ risk from space radiation. Naturwissenschaften.

[22] Chancellor J.C., Scott G.B., Sutton J.P. (2014). Space Radiation: The Number One Risk to Astronaut Health beyond Low Earth Orbit. Life.

[23] Foray N., Bourguignon M., Hamada N. (2016). Individual response to ionizing radiation. Mutat. Res. Mutat. Res..

[24] Wilson J.W., Cucinotta F.A., Shinn J.L., Simonsen L.C., Dubey R.R., Jordan W.R., Jones T.D., Chang C.K., Kim M.Y. (1999). Shielding from solar particle event exposures in deep space. Radiat. Meas..

[25] Cucinotta F.A., Schimmerling W., Wilson J.W., Peterson L.E., Badhwar G.D., Saganti P.B., Dicello J.F. (2001). Space radiation cancer risks and uncertainties for Mars missions. Radiat. Res..

[26] Cucinotta F.A., Durante M. (2006). Cancer risk from exposure to galactic cosmic rays: Implications for space exploration by human beings. Lancet Oncol..

[27] Cucinotta F.A., Manuel F.K., Jones J., Iszard G., Murrey J., Djojonegro B., Wear M. (2001). Space radiation and cataracts in astronauts. Radiat. Res..

[28] Ainsbury E.A., Barnard S., Bright S., Dalke C., Jarrin M., Kunze S., Tanner R., Dynlacht J.R., Quinlan R.A., Graw J. (2016). Ionizing radiation induced cataracts: Recent biological and mechanistic developments and perspectives for future research. Mutat. Res. Rev..

[29] Berthel E., Pujo-Menjouet L., Le Reun E., Sonzogni L., Al-Choboq J., Chekroun A., Granzotto A., Devic C., Ferlazzo M.L., Pereira S. (2023). Toward an Early Diagnosis for Alzheimer’s Disease Based on the Perinuclear Localization of the ATM Protein. Cells.

[30] Al-Choboq J., Mathis T., Restier-Verlet J., Sonzogni L., El Nachef L., Granzotto A., Bourguignon M., Foray N. (2023). The Radiobiological Characterization of Human and Porcine Lens Cells Suggests the Importance of the ATM Kinase in Radiation-Induced Cataractogenesis. Cells.

[31] Pereda-Loth V., Franceries X., Afonso A.S., Ayala A., Eche B., Ginibrière D., Gauquelin-Koch G., Bardiès M., Lacoste-Collin L., Courtade-Saïdi M. (2018). An innovative in vitro device providing continuous low doses of γ-rays mimicking exposure to the space environment: A dosimetric study. Life Sci. Space Res..

[32] Granzotto A., Benadjaoud M.A., Vogin G., Devic C., Ferlazzo M.L., Bodgi L., Pereira S., Sonzogni L., Forcheron F., COPERNIC Project Investigators (2016). Influence of Nucleoshuttling of the ATM Protein in the Healthy Tissues Response to Radiation Therapy: Toward a Molecular Classification of Human Radiosensitivity. Int. J. Radiat. Oncol. Biol. Phys..

[33] Foray N., Marot D., Gabriel A., Randrianarison V., Carr A.M., Perricaudet M., Ashworth A., Jeggo P. (2003). A subset of ATM- and ATR-dependent phosphorylation events requires the BRCA1 protein. EMBO J..

[34] Grote S.J., Joshi G.P., Revell S.H., Shaw C.A. (1981). Observations of Radiation-induced Chromosome Fragment Loss in Live Mammalian Cells in Culture, and Its Effect on Colony-forming Ability. Int. J. Radiat. Biol. Relat. Stud. Phys. Chem. Med..

[35] Le Reun E., Bodgi L., Granzotto A., Sonzogni L., Ferlazzo M.L., Al-Choboq J., El-Nachef L., Restier-Verlet J., Berthel E., Devic C. (2022). Quantitative Correlations between Radiosensitivity Biomarkers Show That the ATM Protein Kinase Is Strongly Involved in the Radiotoxicities Observed after Radiotherapy. Int. J. Mol. Sci..

[36] Fenech M. (2000). The in vitro micronucleus technique. Mutat. Res. Mol. Mech. Mutagen..

[37] Viau M., Sonzogni L., Ferlazzo M.L., Berthel E., Pereira S., Bodgi L., Granzotto A., Devic C., Fervers B., Charlet L. (2021). DNA Double-Strand Breaks Induced in Human Cells by Twelve Metallic Species: Quantitative Inter-Comparisons and Influence of the ATM Protein. Biomolecules.

[38] Rothkamm K., Löbrich M. (2003). Evidence for a lack of DNA double-strand break repair in human cells exposed to very low x-ray doses. Proc. Natl. Acad. Sci. USA.

[39] Schulte-Frohlinde D. (1986). Mechanism of radiation-induced strand break formation in DNA and polynucleotides. Adv. Space Res. Off. J. Comm. Space Res. COSPAR.

[40] Berthel E., Foray N., Ferlazzo M.L. (2019). The Nucleoshuttling of the ATM Protein: A Unified Model to Describe the Individual Response to High- and Low-Dose of Radiation?. Cancers.

[41] Berthel E., Ferlazzo M.L., Devic C., Bourguignon M., Foray N. (2019). What Does the History of Research on the Repair of DNA Double-Strand Breaks Tell Us?—A Comprehensive Review of Human Radiosensitivity. Int. J. Mol. Sci..

[42] Chen J.-H., Hales C.N., Ozanne S.E. (2007). DNA damage, cellular senescence and organismal ageing: Causal or correlative?. Nucleic Acids Res..

[43] Schumacher B., Pothof J., Vijg J., Hoeijmakers J.H.J. (2021). The central role of DNA damage in the ageing process. Nature.

[44] Giovanetti A., Tortolici F., Rufini S. (2020). Why Do the Cosmic Rays Induce Aging?. Front. Physiol..

[45] Guo Z., Zhou G., Hu W. (2022). Carcinogenesis induced by space radiation: A systematic review. Neoplasia.

[46] Kim M.-H.Y., George K.A., Cucinotta F.A. (2006). Evaluation of skin cancer risk for lunar and Mars missions. Adv. Space Res..

[47] Peterson L.E., Pepper L.J., Hamm P.B., Gilbert S.L. (1993). Longitudinal Study of Astronaut Health: Mortality in the Years 1959–1991. Radiat. Res..

[48] Di Trolio R., Di Lorenzo G., Fumo B., Ascierto P.A. (2015). Cosmic radiation and cancer: Is there a link?. Future Oncol. Lond. Engl..

[49] Meier M.M., Matthiä D. (2017). Assessment of the skin dose for aircrew. J. Radiol. Prot. Off. J. Soc. Radiol. Prot..

[50] Little M.P., Wakeford R., Borrego D., French B., Zablotska L.B., Adams M.J., Allodji R., de Vathaire F., Lee C., Brenner A.V. (2018). Leukaemia and myeloid malignancy among people exposed to low doses (<100 mSv) of ionising radiation during childhood: A pooled analysis of nine historical cohort studies. Lancet Haematol..

[51] Little M.P., Pawel D., Misumi M., Hamada N., Cullings H.M., Wakeford R., Ozasa K. (2020). Lifetime Mortality Risk from Cancer and Circulatory Disease Predicted from the Japanese Atomic Bomb Survivor Life Span Study Data Taking Account of Dose Measurement Error. Radiat. Res..

[52] Cucinotta F.A., Kim M.-H.Y., Chappell L.J., Huff J.L. (2013). How Safe Is Safe Enough? Radiation Risk for a Human Mission to Mars. PLoS ONE.

[53] Aleci C. (2020). From international ophthalmology to space ophthalmology: The threats to vision on the way to Moon and Mars colonization. Int. Ophthalmol..

[54] Blakely E.A., Chang P.Y. (2007). A review of ground-based heavy ion radiobiology relevant to space radiation risk assessment: Cataracts and CNS effects. Adv. Space Res..

[55] Longnecker D.E., Manning F.J., Worth M.H., Institute of Medicine (US) Committee on the Longitudinal Study of Astronaut Health (US° Committee on the longitudinal Study of Astronaut Health (2004). Introduction. Review of NASA’s Longitudinal Study of Astronaut Health.

[56] Longnecker D.E., Manning F.J., Worth M.H., Institute of Medicine (US) Committee on the Longitudinal Study of Astronaut Health (US° Committee on the longitudinal Study of Astronaut Health (2004). Findings to Date. Review of NASA’s Longitudinal Study of Astronaut Health.

[57] NEA (2012). 2011 NEA Annual Report.

[58] Pinsky L.S., Osborne W.Z., Bailey J.V., Benson R.E., Thompson L.F. (1974). Light flashes observed by astronauts on Apollo 11 through Apollo 17. Science.

[59] McNulty P.J., Pease V.P., Bond V.P. (1976). Comparison of the Light Flash Phenomena Observed in Space and in Laboratory Experiments.

[60] Bidoli V., Casolino M., De Pascale M.P., Furano G., Morselli A., Narici L., Picozza P., Reali E., Sparvoli R., Galper A.M. (2000). Study of cosmic rays and light flashes on board Space Station MIR: The SilEye experiment. Adv. Space Res. Off. J. Comm. Space Res. COSPAR.

[61] Bidoli V., Casolino M., De Pascale M.P., Furano G., Minori M., Morselli A., Narici L., Picozza P., Reali E., Sparvoli R. (2002). The Sileye-3/Alteino experiment for the study of light flashes, radiation environment and astronaut brain activity on board the International Space Station. J. Radiat. Res..

[62] Avdeev S., Bidoli V., Casolino M., De Grandis E., Furano G., Morselli A., Narici L., De Pascale M.P., Picozza P., Reali E. (2002). Eye light flashes on the Mir space station. Acta Astronaut..

[63] Iwamoto J., Takeda T., Sato Y. (2005). Interventions to prevent bone loss in astronauts during space flight. Keio J. Med..

[64] Farley A., Gnyubkin V., Vanden-Bossche A., Laroche N., Neefs M., Baatout S., Baselet B., Vico L., Mastrandrea C. (2020). Unloading-Induced Cortical Bone Loss is Exacerbated by Low-Dose Irradiation During a Simulated Deep Space Exploration Mission. Calcif. Tissue Int..

[65] Vico L., Hargens A. (2018). Skeletal changes during and after spaceflight. Nat. Rev. Rheumatol..

[66] Axpe E., Chan D., Abegaz M.F., Schreurs A.-S., Alwood J.S., Globus R.K., Appel E.A. (2020). A human mission to Mars: Predicting the bone mineral density loss of astronauts. PLoS ONE.

[67] Restier-Verlet J., Joubert A., Ferlazzo M.L., Granzotto A., Sonzogni L., Al-Choboq J., El Nachef L., Le Reun E., Bourguignon M., Foray N. (2023). X-rays-Induced Bystander Effect Consists in the Formation of DNA Breaks in a Calcium-Dependent Manner: Influence of the Experimental Procedure and the Individual Factor. Biomolecules.

[68] Bachelet J.-T., Granzotto A., Ferlazzo M., Sonzogni L., Berthel E., Devic C., Foray N. (2020). First Radiobiological Characterization of Skin and Bone Cells from A Patient Suffering from the PI3KCA-Related Overgrowth Spectrum (PROS) Syndrome. Arch. Clin. Med. Case Rep..

[69] Darby S.C., Ewertz M., McGale P., Bennet A.M., Blom-Goldman U., Brønnum D., Correa C., Cutter D., Gagliardi G., Gigante B. (2013). Risk of ischemic heart disease in women after radiotherapy for breast cancer. N. Engl. J. Med..

[70] Taylor C., Dodwell D., McGale P., Hills R.K., Berry R., Bradley R., Braybrooke J., Clarke M., Gray R., Holt F. (2023). Radiotherapy to regional nodes in early breast cancer: An individual patient data meta-analysis of 14 324 women in 16 trials. Lancet.

[71] Tang W., Zhou M., Dorsey T.H., Prieto D.A., Wang X.W., Ruppin E., Veenstra T.D., Ambs S. (2018). Integrated proteotranscriptomics of breast cancer reveals globally increased protein-mRNA concordance associated with subtypes and survival. Genome Med..

[72] Reynolds R.J., Day S.M. (2017). Mortality Due to Cardiovascular Disease Among Apollo Lunar Astronauts. Aerosp. Med. Hum. Perform..

[73] Krittanawong C., Isath A., Kaplin S., Virk H.U.H., Fogg S., Wang Z., Shepanek M., Scheuring R.A., Lavie C.J. (2023). Cardiovascular disease in space: A systematic review. Prog. Cardiovasc. Dis..

[74] Dai Z., Lei X., Yang C., Zhao L., Lu L., Li Y. (2019). Systematic biomedical research of the NASA Twins Study facilitates the hazard risk assessment of long-term spaceflight missions. Protein Cell.

[75] Garrett-Bakelman F.E., Darshi M., Green S.J., Gur R.C., Lin L., Macias B.R., McKenna M.J., Meydan C., Mishra T., Nasrini J. (2019). The NASA Twins Study: A multidimensional analysis of a year-long human spaceflight. Science.

[76] Witze A. (2019). Astronaut twins study spots subtle genetic changes caused by space travel. Nature.

[77] Luxton J.J., Bailey S.M. (2021). Twins, Telomeres, and Aging-in Space!. Plast. Reconstr. Surg..

[78] Schmidt M.A., Meydan C., Schmidt C.M., Afshinnekoo E., Mason C.E. (2020). The NASA Twins Study: The Effect of One Year in Space on Long-Chain Fatty Acid Desaturases and Elongases. Lifestyle Genom..

[79] Roti Roti J.L. (1982). Heat-induced cell death and radiosensitization: Molecular mechanisms. Natl. Cancer Inst. Monogr..

[80] Roti Roti J.L., Henle K.J., Winward R.T. (1979). The kinetics of increase in chromatin protein content in heated cells: A possible role in cell killing. Radiat. Res..

[81] Donaubauer A.-J., Deloch L., Becker I., Fietkau R., Frey B., Gaipl U.S. (2020). The Influence of Radiation on Bone and Bone Cells-Differential Effects on Osteoclasts and Osteoblasts. Int. J. Mol. Sci..

[82] Mazel P., Foray N., Pujo-Menjouet L. A Mathematical Model to Describe the Formation of Perinuclear ATM Crown and the Effect of Irradiation and Antioxidants in Cells Affected by Alzheimer’s Disease. Lecture Notes on Mathmatical Modeling in Life-Sciences. Springer, Accepted. http://math.univ-lyon1.fr/~pujo/publications-pujo-menjouet.html.

