# Supplementary material for: Accelerated Aging Effects Observed In Vitro after an Exposure to Gamma-Rays Delivered at Very Low and Continuous Dose-Rate Equivalent to 1–5 Weeks in International Space Station
Source: Cells. 2024 Oct 15;13(20):1703. doi: 10.3390/cells13201703 (PMC11506070; doi:10.3390/cells13201703)
Supplement: Supplementary file 1 [file cells-13-01703-s001.zip › cells-3253542-supplementary.pdf]

Accelerated Aging Effects Observed In Vitro after an Exposure to Gamma-Rays Delivered at Very Low and Continuous Dose-Rate Equivalent to 1–5 Weeks in International Space Station

Juliette Restier-Verlet, Mélanie L. Ferlazzo, Adeline Granzotto, Joëlle Al-Choboq, Camélia Bellemou, Maxime Estavoyer, Florentin Lecomte, Michel Bourguignon, Laurent Pujo-Menjouet and Nicolas Foray *

Mathematical model

The stress-induced dissociation parameter is represented by $\boldsymbol{g_c}$. However, HDC are supposed to be associated with an acidification of cytoplasm inducing a stress factor $\boldsymbol{S}$. We model $\boldsymbol{g_A}$ as follows,

$$\boldsymbol{g_A}(\boldsymbol{S}) = \boldsymbol{g_c} + \boldsymbol{\alpha S},\ \boldsymbol{\alpha} \in \mathbb{R}^+. \qquad \textbf{(S1)}$$

Cytoplasmic ATM dimers ($\boldsymbol{D_c}$) are produced by the cell at the rate $\boldsymbol{\lambda}$**,** supposed to be constant in the model. It naturally degrades itself at the rate $\boldsymbol{d_0}$. It can monomerize through the stress $\boldsymbol{g_c}$. Dimers also come from the dimerization of ATM monomers. The constant rate at which ATM monomers tend to reform more stable ATM dimers is $\boldsymbol{k_1} \in \mathbb{R}^+$. The coefficient $\frac{1}{2}$ comes from one ATM dimer giving two ATM monomers.

Cytoplasmic ATM monomers ($\boldsymbol{M_C}$) come from the monomerization of $\boldsymbol{D_C}$. They also dimerize. It goes around the nucleus through the rate

$$\boldsymbol{k}_2(\boldsymbol{D_A}) = \boldsymbol{b}_2 \frac{\boldsymbol{a}_2^{\boldsymbol{n}_2}}{\boldsymbol{a}_2^{\boldsymbol{n}_2} + \boldsymbol{D}_A^{\boldsymbol{n}_2}},\ \boldsymbol{a}_2, \boldsymbol{b}_2, \boldsymbol{n}_2 \in \mathbb{R}^+. \quad \textbf{(S2)}$$

The more ATM dimers are around the nucleus, the fewer ATM monomers will go around the nucleus.

ATM monomers around the nucleus ($\boldsymbol{M_A}$) can exchange with $\boldsymbol{M_C}$ at the rate described above. Monomers can go inside the nucleus at the rate:

$$\boldsymbol{k}_3(\boldsymbol{D_A}) = \boldsymbol{b}_3 \frac{\boldsymbol{a}_3^{\boldsymbol{n}_3}}{\boldsymbol{a}_3^{\boldsymbol{n}_3} + \boldsymbol{D}_A^{\boldsymbol{n}_3}},\ \boldsymbol{a}_3, \boldsymbol{b}_3, \boldsymbol{n}_3 \in \mathbb{R}^+. \quad \textbf{(S3)}$$

The amount of $\boldsymbol{M_A}$ that can pass through the nuclear membrane is inversely proportional to the amount of ATM dimers in the PC. For radiosensitive individuals, ATM monomers will gather with X-proteins to create ATM-X protein complexes at the constant rate $\boldsymbol{k_4} \in \mathbb{R}^+$. Monomers can also form ATM dimers at the rate:

$$\boldsymbol{k}_5(\boldsymbol{C_A}) = \frac{\boldsymbol{b}_5 \boldsymbol{C}_A^{\boldsymbol{n}_5}}{\boldsymbol{a}_5^{\boldsymbol{n}_5} + \boldsymbol{C}_A^{\boldsymbol{n}_5}},\ \boldsymbol{a}_5, \boldsymbol{b}_5, \boldsymbol{n}_5 \in \mathbb{R}^+. \quad \textbf{(S4)}$$

The numbers of ATM-X protein complexes influence the dimerization of ATM. Both formations of complexes or dimers can be reversed with the stress $\boldsymbol{g_A}$.

ATM dimers around the nucleus ($\boldsymbol{D_A}$) come from the dimerization of $\boldsymbol{M_A}$ and can be monomerized in $\boldsymbol{M_A}$ at the rates described above.

X proteins ($\boldsymbol{A}$) create ATM-X complexes at the rate $\boldsymbol{k_4} \in \mathbb{R}^+$. It goes from complex to its initial state at the rate $\boldsymbol{g_A}$. ATM-X complex ($\boldsymbol{C_A}$) comes from the association of $\boldsymbol{M_A}$ and $\boldsymbol{A}$, and can be dissociated at the rates described above. Monomer of ATM inside the nucleus ($\boldsymbol{M_N}$) comes from the PC at the rate $\boldsymbol{k_3}$. It also degrades at the rate $\boldsymbol{d_1}$.

The radius of the nucleus is represented by $\boldsymbol{R}$. It increases only if the amount of ATM in the PC exceeds the threshold value $\boldsymbol{K}$. When the threshold is reached, the radius increases proportionally to $\boldsymbol{r_1}$. The function $\boldsymbol{k_7}$ is a Hill function delaying size increase.

$$\boldsymbol{k_7(C)} = \frac{\boldsymbol{b_c(C-K)^{n_c}}}{\boldsymbol{a_c^{n_c}+(C-K)^{n_c}}}, \quad \boldsymbol{a_c, b_c, n_c} \in \mathbb{R}^+, \quad \textbf{(S5)}$$

Where $\boldsymbol{C}$ represents the number of all ATM forming the PC. ATM is found in monomers $\boldsymbol{M_A}$, dimers $\boldsymbol{D_A}$, and complexes $\boldsymbol{C_A}$. The corresponding relationship is :

$$\boldsymbol{C = M_A + 2D_A + C_A}. \qquad \textbf{(S6)}$$

The stress produced by the acidification of the cytoplasm through the enlargement of the nucleus is represented by $\boldsymbol{S}$. It increases only if the amount of ATM in the PC exceeds the threshold value $\boldsymbol{K}$. When the threshold is reached, the radius increases proportionately to $\boldsymbol{s_1}$.

Table S1: Parameters used for the numerical simulation

**Parameters**	**Value**
λ	15
d_0	0.05
d_1	0.25
K	80
Parameters of $\boldsymbol{g_A}$ and $\boldsymbol{g_c}$	
g_c	0.052
α	0.001
Parameter of $\boldsymbol{k_1}$	
k_1	0.01
Parameters of $\boldsymbol{k_2}$	
a_2	400
b_2	2
n_2	10
Parameters of $\boldsymbol{k_3}$	
a_3	80
b_3	0.1
n_3	10
Parameter of $\boldsymbol{k_4}$	
k_4	0.05
Parameters of $\boldsymbol{k_5}$	
a_5	20
b_5	40
n_5	10
Parameters of $\boldsymbol{k_7}$	
a_C	30
b_C	0.5

n_C	2
Parameters of $\boldsymbol{R}$ and $\boldsymbol{S}$	
r_1	5
s_1	5
Initial conditions	
D_{C_0}	50
M_{C_0}	0
M_{A_0}	0
M_{N_0}	0
A_0	30
C_{A_0}	0
D_{A_0}	0
R_0	10
S_0	0

We can see that the distribution of the percentage of crowns over time is Gaussian. HDC Gaussian distribution is shifted, as shown in Figure 2. Once the PC is formed, DNA unfolds and the radius of the nucleus progressively increases. When the radius is large enough for ATM to penetrate the nucleus, HDC begins to appear visible in the microscope. For the time being, this explanation for this asynchronous nature remains speculative and requires further investigations. This could be an interpretation for B in Figure 2. For C and D, the larger quantity of HDC could be explained by the accumulation of calcium inside the cell.
